# Phenolic Foam Preparation Using Hydrofluoroolefin Blowing Agents and the Toughening Effect of Polyethylene Glycol

**DOI:** 10.3390/polym16182558

**Published:** 2024-09-10

**Authors:** P. R. Sarika, Paul Nancarrow, Taleb H. Ibrahim

**Affiliations:** Department of Chemical & Biological Engineering, American University of Sharjah, Sharjah P.O. Box 26666, United Arab Emirates; sreghunadh@aus.edu (P.R.S.); italeb@aus.edu (T.H.I.)

**Keywords:** phenolic foam, blowing agents, hydrofluoroolefin, polyethylene glycol, mechanical properties

## Abstract

In this work, a new class of fourth-generation, zero ozone depletion potential, hydrofluoroolefin-based blowing agents were used to prepare phenolic foam. While hydrofluoroolefin blowing agents have been used previously to prepare polyurethane foams, few studies have been reported on their use in phenolic foams. We introduce an effective method for foam preparation using two low-boiling blowing agents, cis-1,1,1,4,4,4-hexafluoro-2-butene and trans-1,1,1,4,4,4-hexafluoro-2-butene, and their combinations with hexane. Traditionally, phenolic foams have been prepared using chlorofluorocarbons and hydrochlorofluorocarbons, which can have harmful effects on the environment due to their high ozone depletion potential or global warming potential. Conductor-like screening model for real solvents (COSMO-RS) modeling studies were performed to understand the effects of different blowing agent combinations on their boiling points. A series of phenolic foams were prepared by varying the concentration of the hydrofluoroolefin and the hydrofluoroolefin–hexane blowing agent combinations. The concentrations of the surfactant, Agnique CSO 30, and the toughening agent, polyethylene glycol, were also varied to yield a formulation with the optimal properties. The foams formulated with the hydrofluoroolefin–hexane mixture displayed a higher compressive strength and a lower thermal conductivity than those prepared with either hydrofluoroolefin or hexane alone. The cell microstructure of all the foams was examined using scanning electron microscopy. By introducing flexible chains into the resin matrix, PEG facilitates proper distribution of hydrofluoroolefin–hexane blowing agents and other reagents and thereby increases the mechanical strength of the foam.

## 1. Introduction

Insulation materials are widely used for energy conservation in industrial facilities, cold storage, refrigerated rooms, and buildings as suspended ceilings and partitioning materials [1,2]. They can also be used to insulate ships, life jackets, buoyancy blocks, and several marine types of equipment. Phenolic foams have certain advantages over other insulation materials due to their very low thermal conductivity, low flammability, low density, and good chemical and heat resistance. However, phenolic foams also suffer from some problems, particularly related to the difficulty handling them due to their poor mechanical strength. In many industrial processes, materials with less effective thermal insulation properties are selected over phenolic foams due to the need for easier handling during processing and a higher strength in end use. Over the years, several modifications have been made to phenolic foams to improve their mechanical properties via the addition of toughening agents or via chemical modification of the resin structure by substituting phenols and formaldehyde with biobased phenols and aldehydes to obtain more environmentally friendly and efficient foams [3,4,5,6].

The foaming process in phenolic foams can be achieved either using physical or chemical foaming agents. The insulating properties of the phenolic foam mainly depend on the cell structure induced by the blowing agents [7,8]. Foams with closed cells are mainly used for insulating applications [9]. The blowing agents stay trapped in closed cells, and their thermal conductivity is one of the most important factors in defining the insulating properties of the foam. A commonly used foaming method is physical foaming with liquid blowing agents with a low boiling point [10]. Chlorofluorocarbons (CFCs) are first-generation blowing agents which were widely used in phenolic and polyurethane foams until the early 1980s [11]. The use of CFCs has been regulated and restricted worldwide under the Montreal Protocol and its amendments due to their destructive effect on the ozone layer [12]. Hydrochlorofluorocarbons (HCFCs) were considered second-generation blowing agents and used as replacements for CFCs due to the fast degradation of HCFCs in lower atmospheric levels, which resulted in a lower impact on the ozone layer [13]. Though HCFCs have a lower ozone depletion potential (ODP), their usage was banned in Europe in 2004 [14], and the Montreal Protocol in 2007 decided on an immediate phase-out due to their high global warming potential (GWP) [11,15]. Cost-effective and non-polluting blowing agents such as pentane blends [16,17] or isomers, cyclopentane [3], and hydrofluorocarbons (HFCs, third-generation blowing agents) are widely used in the current production of phenolic and polyurethane foams. However, HFCs were selected as regulated substances in the 2016 Montreal Convention due to their global warming potential [18]. Increasing environmental awareness has led to the development of new blowing agents, based on hydrofluoroolefins (HFOs), with zero ODP and a low GWP. HFOs are considered the fourth-generation blowing agents [19].

This work describes the use of fourth-generation HFO blowing agents such as cis-1,1,1,4,4,4-hexafluoro-2-butene (HFO-1336mzz (Z)) and trans-1,1,1,4,4,4-hexafluoro-2-butene (HFO-1336mzz (E)) in phenolic foams and their effects on the properties of the foams. HFO-1336mzz (Z) and HFO-1336mzz (E) are non-flammable, fourth-generation blowing agents with zero ODP, a low GWP, and very low vapor thermal conductivity. HFO blowing agents have been used in the preparation of polyurethane/polyisocyanurate foams [20], but few researchers have reported their use in phenolic foams.

While there are several studies on the effect of different types of blowing agents on the cellular structure and mechanical properties of polyurethane foams [21,22,23,24], studies on various properties of phenolic foams using different blowing agents are limited. Saz-Orozco et al. [25] studied the effects of the wood flour weight fraction and n-pentane blowing agent concentrations on various properties, such as the density, compressive properties, and morphology, of phenolic foams. Limited reports are available on the use of HFO blowing agents in phenolic foam production [26,27,28]. Hence, this study focuses on the application and optimization of HFO blowing agents in the foaming of phenolic foams and their effects on properties such as their compressive strength, foam morphology, and thermal conductivity.

The low mechanical strength of phenolic foams is also addressed in this work. Nanoparticles, biopolymers, wood flour, and microparticles have already been used to impart mechanical strength to phenolic foams [17,29,30,31,32]. The introduction of polymers or long-chain molecules into the resin matrix increases their mechanical strength by adding flexibility to the resin structure, either by physical or chemical interactions or by intermolecular bonding. In this work, polyethylene glycol was used as a combined toughening agent and surfactant, along with HFO blowing agents, resulting in a synergetic effect on the mechanical and insulating properties. Sui et al. [33] studied the toughening effect of phosphorous modified polyethylene glycol (PPEG) on phenolic foams. The incorporation of PPEG was found to increase the mechanical strength of the foam. Hu et al. [34] demonstrated the effect of PEG on the microstructure, thermal stability, and fire properties of phenol–urea–formaldehyde foam. PEG improved the toughening of the phenolic foam by forming hydrogen bonds with the OH groups in the phenolic resin. The resulting long PEG–O–HO–resin bonds introduced toughness into the foam by imparting flexibility to the chains in bearing high applied loads [35].

In this study, a new formulation was prepared with HFO blowing agents which had zero ODP and a low GWP. The boiling points of different blowing agent combinations were initially studied using COSMO-RS modeling. A series of PFs were prepared using HFO-1336mzz (Z) and HFO-1336mzz (E) and mixtures of them with hexane. The formulation which gave the best mechanical and thermal properties was selected for studying the effects of PEG. Various additional formulations were prepared by changing the concentration of PEG, and the impact on their compressive strength and morphology was analyzed.

## 2. Experimental Details

### 2.1. Materials

Phenol (detached crystal, CAS No: 108-95-2) was purchased from Fisher, Loughborough, UK. The formaldehyde solution (37%, CAS No: 50-00-0), sodium hydroxide (CAS No:1310-73-2), polyethylene glycol 600 (CAS No: 25322-68-3), and n-hexane (CAS No: 110-54-3) were obtained from Merck, Damstadt, IN, USA. Methane sulfonic acid (Lutropur MSA, CAS No: 75-75-2) and Agnique CSO 30 (Ethoxylate castor oil, CAS No: 61791-12-6) were purchased from BASF, Ludwigshafen, Germany. Cis-1,1,1,4,4,4-hexafluoro-2-butene (HFO-1336mzz (Z), CAS No: 692-49-9) and trans-1,1,1,4,4,4-Hexafluoro-2-butene (HFO-1336mzz (E), CAS No: 66711-86-2) were obtained from Chemours, Delaware, PA, USA, under the commercial names Opteon 1100 and Opteon 1150, respectively. 

### 2.2. Preparation of the Phenolic Resin (PR)

Phenol formaldehyde resin was prepared with a phenol and formaldehyde ratio of 1:1.5 using a two-step formaldehyde addition method [36,37]. Resin synthesis is an exothermic reaction; hence, a two-step formaldehyde addition method involving a gradual increase in the reaction temperature was adopted to control the reaction and the viscosity of the resin. A longer reaction time was preferred to ensure the complete conversion of the reactants. In the first stage, phenol (1 mol) was melted at 40–45 °C in a three-neck round-bottom flask (1 L) kept in an oil bath equipped with a thermometer, a reflux condenser, and a dropping funnel. Sodium hydroxide aqueous solution (15 mL, 50% *w*/*v*) was added to the molten phenol to adjust the pH to 9 at the same temperature and reacted for 30 min. The temperature was increased to 60 °C, and the first 80% of the total formaldehyde solution was added dropwise through a dropping funnel and reacted for 1 h at the same temperature. Before the second-stage addition of formaldehyde, 5 mL NaOH was added to the reaction mixture to adjust the pH, and the temperature was increased to 85 °C. The remaining 20% of the formaldehyde solution was added when the temperature reached 70 °C. After its addition, the reaction was continued for a further 2.5 h at 85 °C. The resin was cooled down to 50–60 °C after the reaction and distilled to obtain the desired viscosity. Distillation was necessary to remove the excess water produced as a byproduct of the resin’s formation.

### 2.3. Preparation of the Phenolic Foam (PF)

The foams were prepared by mixing the phenolic resin, surfactants, blowing agents, and catalysts and subsequently curing and post curing them in a closed mold. The mixing was performed in an ice bath to prevent premature evaporation of the low-boiling-point blowing agents. Initially, the synthesized resin, Agnique CSO 30, and PEG were mixed thoroughly using a hand blender for 2–3 min. Then, different combinations of the blowing agents HFO-1336mzz (Z) and HFO-1336mzz (E) and the HFO–hexane mixture were added to the resin–surfactant mixture and mixed for one minute. Finally, the methane sulfonic acid catalyst was added, and the mixture was thoroughly blended, immediately poured into a preheated closed aluminum foil mold, and cured at 80 °C for an hour in an oven. Post curing was performed at 60 °C for 2 h.

### 2.4. Characterization of the Phenolic Resin

The physical properties of the resin, such as its pH, viscosity, and solid content, were analyzed. The resin’s viscosity was measured using an NDJ-8S rotational viscometer (Chincan trade.com, Shanghai, China) at room temperature (25 °C). The solid content of the resin was determined in accordance with ASTM standard D4426-01 [38]. The pH of the resin was measured using a digital pH meter (Bluelab, Tauranga, New Zealand).

### 2.5. Characterization of the Phenolic Foam

The physical, mechanical, and thermal properties of the prepared foams were analyzed. The apparent density of all the foams was calculated according to ASTM D1622. The morphology of the PFs was visualized using a VEGA 3 (TESCAN, Brno–Kohoutovice, Czech Republic) scanning electron microscope (SEM) operated with an accelerating voltage of 20 kV. The mean cell size and cell size distributions of each PF were calculated from at least 100 cells in the SEM images using ImageJ software (version 1.54d). The compressive strength of the foams was measured in accordance with ASTM D1621 [39] on a universal testing machine (Instron, MA, USA). The compressive strength of the foams was determined from the stress–strain curve as the maximum value when the strain was <10%. A TCi thermal conductivity analyzer (C-Therm, New Brunswick, Canada) was used to measure the thermal conductivity of the foams. The measurements were performed at 25 °C, and the results were reported as an average of three samples with a size of 30 × 30 × 30 mm^3^. The same samples were used for the apparent density measurements, compression, and the thermal conductivity analysis. The thermal behavior of the foams was analyzed on a STA 6000 (Perkin Elmer, MA, USA) thermo-analyzer instrument. Samples with a weight of 5–10 mg were heated from room temperature to 900 °C at a scan rate of 10 °C min^−1^ in nitrogen (20 mL/min).

### 2.6. COSMO-RS Modeling

COSMO-RS modeling was used to predict the phase equilibria of the different HFO–alkane blowing agent combinations. Initially, the TURBOMOLE quantum chemistry package [40] was used to perform COSMO calculations on each individual molecule. The calculations were carried out at the density functional theory (DFT) level, with the Becke–Perdew (BP) functional [41,42] and a triple-valence polarized basis set (TZVP) [43]. Where applicable, optimized radii [44] were used in the COSMO calculations (H = 1.30, C = 2.00, O = 1.72, F = 1.72).

Next, for the COSMO-RS statistical thermodynamics modeling, the COSMO files generated in the previous step were imported into the COSMOthermX software (version 19.0). σ-surfaces and σ-profiles were generated for each component, followed by simulations on mixtures of the HFO and alkane blowing agents to generate temperature–composition (T-x-y) diagrams.

## 3. Results and Discussion

All of the phenolic foams described in this work were prepared from resol-type phenolic resin synthesized at a formaldehyde:phenol ratio of 1.5. The resin’s properties, especially its viscosity, solid content, and pH, play a critical role in the foam-making process, as they influence the distribution of the blowing agents, surfactants, and catalysts in the foaming resin and thereby the properties of the resulting foam [45,46]. Highly viscous resin prevents proper mixing, which results in a dense foam, whereas a low-viscosity resin causes an uncontrolled expansion of the resin mixture, and the corresponding foam will have a very low density and undesired properties. Hence, an appropriate viscosity is essential for uniform mixing of the foaming reagents in the resin [31]. Therefore, in this work, resin with a viscosity in the range between 8000 and 9000 mPa.s was used to make the various phenolic foams. The solid content of the resin was 79–82%, and its pH was 9.6.

**Figure 1:**
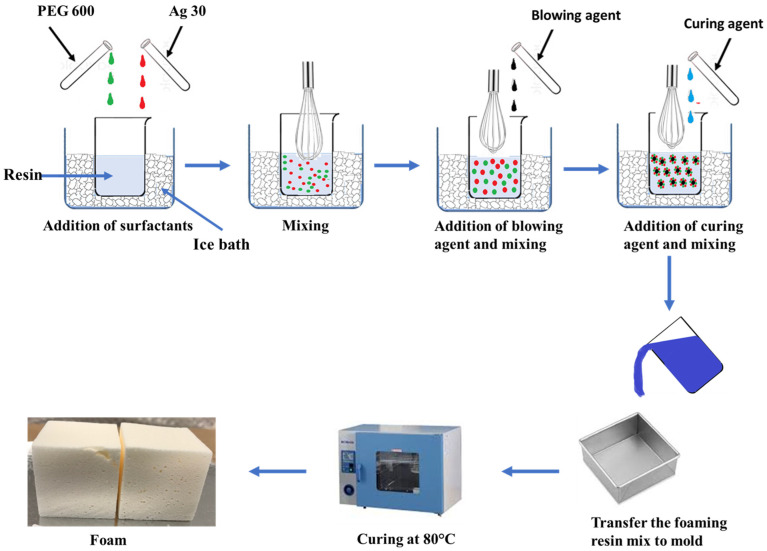
Schematic diagram of the foaming process.

Phenolic foam was prepared by mixing the surfactants, blowing agents, and catalysts at appropriate ratios into the resin and subsequently curing the foaming resin at a suitable temperature, either in an open or closed mold, depending upon the application. All of the PFs listed in this work were prepared via a four-step process. A schematic representation of the foaming process is shown in Figure 1. Mixing of all the reagents into the resin was performed in an ice bath to prevent premature boiling of the HFO-1336mzz blowing agents.

**Figure 2:**
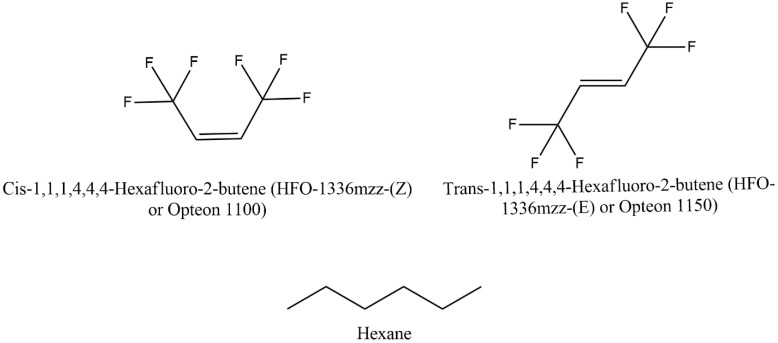
Structures of the blowing agents used in this work.

In the first step, the surfactants Agnique CSO 30 and PEG were mixed into the resin for a minute. In the second step, the blowing agent, HFO-1336mzz (E), HFO-1336mzz (Z), or the HFO–hexane mixture, was mixed into the resin–surfactant mixture as quickly as possible to avoid any loss. In the third step, the catalyst was mixed rapidly, and immediately, the resin mixture was poured into a preheated mold. In the final step, curing and post curing were performed. The final properties of the foam are affected by the selection of the reagents used in the synthesis, the relative proportions, and the processing conditions. Several studies have been reported in the literature on evaluating the effect of various parameters on the properties of PFs [45,47,48,49]. In this work, a series of phenolic foams were prepared to study the impact of incorporating an HFO blowing agent on the foam’s properties. Two types of HFO blowing agents were used in this work, and their properties and structures are listed in Table 1 and Figure 2, respectively.

The foams formulated with HFO-1336mzz (Z), HFO-1336mzz (E), and mixtures of them with hexane are listed in Table 2, and their properties are shown in Table 3.

**Figure 3:**
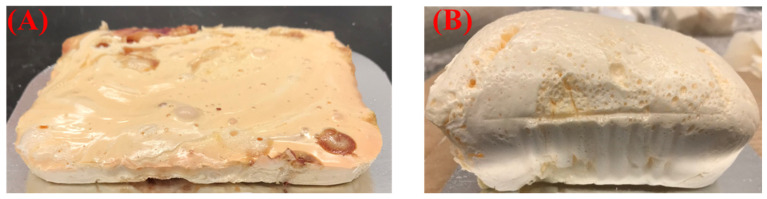
Phenolic foams formulated with (**A**) HFO-1336mzz (E) (PF OP1) and (**B**) HFO-1336mzz (Z) (PF OP2) blowing agents.

The boiling points of HFO-1336mzz (Z), HFO-1336mzz (E), and hexane are 33 °C, 7 °C, and 68.7 °C, respectively. Since the HFO blowing agents have low boiling points, all of the mixing steps in the foam-making process were performed in an ice bath to ensure the maximum availability of the blowing agents within the foam matrix. The foam PF OP1 shown in Figure 3 was formulated with HFO-1336mzz (E) and did not expand fully due to it curing more quickly than all of the other foams. Though the mixing was performed in an ice bath, significant loss of HFO-1336mzz (E) occurred during the mixing step itself; hence, the foam was cured before it could complete the expansion process. PF OP2 formulated with HFO-1336mzz (Z) reached a higher level of expansion compared with PF OP1. However, it still showed a very high density, along with a high thermal conductivity and compressive strength. Since HFO-1336mzz (Z) has a higher boiling point, its loss during the mixing was comparatively less than that of its isomer.

Though HFO-1336mzz blowing agents have the advantages of a low GWP, zero OPD, and low thermal conductivity, the observed processing behavior of the two formulations PF OP1 and PF OP2 indicates that the foam-making process with these blowing agents required special processing arrangements due to their low boiling points, such as refrigeration, to ensure the complete mixing and retention of the reagents in the resin matrix. One possible method for overcoming the rapid loss of low-boiling HFO-1336mzz blowing agents is to use them in combination with other suitable higher-boiling-point blowing agents. COSMO-RS modeling was performed to study the vapor–liquid equilibria of mixtures between the HFO-1336mzz blowing agents and typical hydrocarbons used as blowing agents for phenolic foam manufacturing. The synergistic effects of using combinations of HFO-1336mzz (E) and HFO-1336mzz (Z) with hydrocarbons were also studied by COSMO-RS modeling.

**Figure 4:**
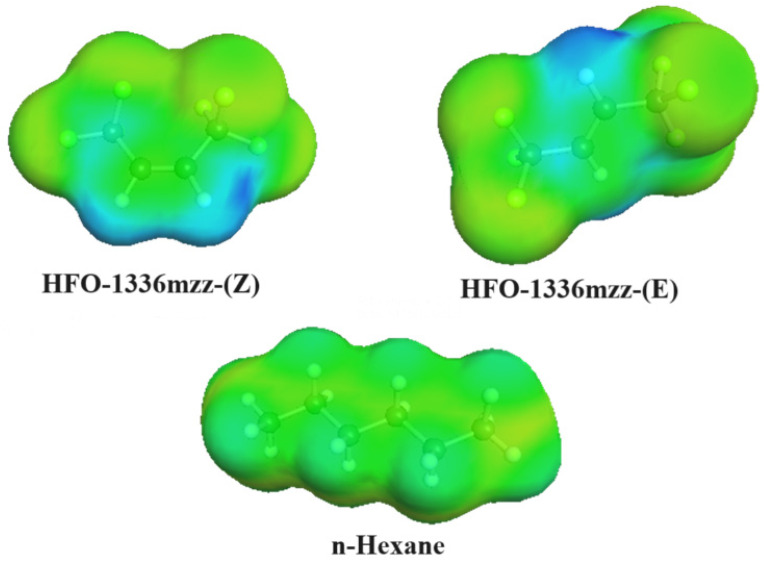
COSMO surfaces for the blowing agents used in this study.

The method involves first carrying out a quantum calculation to optimize the 3D geometric structure and surface charge distribution, known as the COSMO file, for each molecule. The second step is to use the results of these quantum calculations in statistical thermodynamics to predict the phase equilibrium behavior of the mixtures involving these molecules. The COSMO surfaces for each of the molecules used in this study are shown in Figure 4. The green color represents neutral regions, the blue color shows positively charged regions (low electron density), and yellow/red shows regions with a negative charge (high electron density). We can observe that the HFO molecules have a somewhat higher polarity than the non-polar hydrocarbons. This is indicated by the stronger blue and yellow regions versus the almost uniform green color of the hydrocarbons. This dissimilarity between the molecules in terms of their polarity means that these molecules are likely to exhibit non ideal VLE behavior.

**Figure 5:**
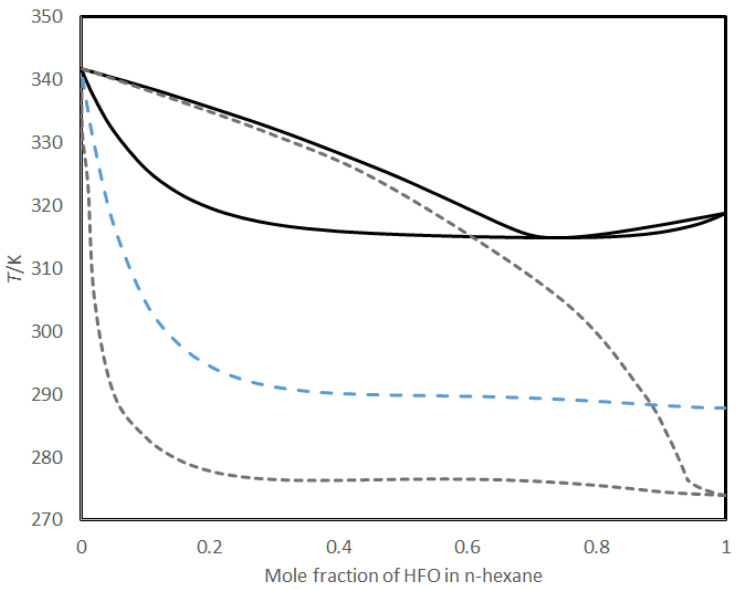
T-x-y diagram for HFO-1336mzz (Z) with n-hexane (black solid lines) and HFO-1336mzz (E) with n-hexane (graydashed lines) and bubble point curve for a 50:50 mixture (mol basis) of HFO-1336mzz (Z) and HFO-1336mzz (E) with n-hexane (blue dashed line).

The predicted VLE data for the HFO blowing agents with n-hexane are shown in Figure 5. Strong deviation from the ideal solution behavior is exhibited in all cases. This is most pronounced for the HFO-1336mzz (E)–hexane mixture, where a small amount of HFO-1336mzz (E) dramatically reduces the bubble point of the mixture. This indicates that a combination of n-hexane with small amounts of HFO-1336mzz (E), or a blend of HFO-1336mzz (E) with HFO-1336mzz (Z), may give a good compromise between ease of handling and the thermal performance of the resulting foams.

Based on the modeling studies and the experimental results for the PF OP1 and PF OP2 formulations, a mixture of HFO and hexane was considered a promising combination of blowing agents. Hence, the PFs from PF OP3 to PF OP10 were formulated using mixtures of HFO-1336mzz (Z) and (E) with hexane (HFO–hexane) to ensure the best balance between their expansion rate and the retention of the blowing agents. The PF prepared with hexane blowing agents showed a moderate expansion rate, and the resulting foam had a density of 55 kg/m^3^ with a low thermal conductivity of 0.036 W/m.K and a compressive strength of 122 kg/m^3^. Since the boiling point of hexane is higher than room temperature, it mixes uniformly into the resin without any evaporative loss, resulting in a stable foam with a low density and thermal conductivity and a high mechanical strength.

The percentages of HFO-1336mzz and hexane were varied such that the total blowing agent content in the resin mixture remained at 7 mL. The standard PF (sample PF) was formulated with 0.053 moles of hexane, whereas PF OP3 and PF OP4 were formulated by replacing 0.008 moles with HFO-1336mzz (Z) and HFO-1336mzz (E), respectively, and the resulting foams had densities of 63 ± 3 and 70 ± 2 kg/m^3^, respectively. The slight variation in density is due to the difference in the boiling point of the HFO–hexane mixture. The bubble point of HFO-1336mzz (E)–hexane (Figure 5) is lower than that of the HFO-1336mzz (Z)–hexane mixture; hence, the foam’s expansion and curing occurred at a faster rate for PF OP4, which resulted in a slight increase in density as compared to PF OP3. In PF OP3, HFO-1336mzz (Z)–hexane evaporates more slowly, which leads to slow expansion and subsequent curing. As a result, the density of PF OP3 was slightly lower than that of PF OP4. PF OP5 was formulated with the HFO–hexane mixture containing an equal molar ratio of the HFO-1336mzz (E) and (Z) blowing agents. The density of the foams increases with an increase in the percentage of HFO-1336mzz in the blowing agent mixture (PF OP6, PF OP7, and PF OP8). This may be due to the reduction in the bubble point with an increase in the mole fraction of the HFO-1336mzz blowing agents. Loss of the blowing agents may occur due to premature evaporation during mixing and also due to the more rapid expansion of the foaming mixture during the foaming process. Hence, the actual amount of blowing agents remaining within the resin matrix for foam expansion was less than the calculated value for the mixtures using higher concentrations of HFO-1336mzz. Consequently, the foams with higher HFO-1336mzz additions show high density due to fast expansion, loss of the blowing agent due to evaporation, and subsequent curing of the foaming resin.

Among the HFO–hexane formulations, PF OP3 and PF OP5 are the most promising, as they have a low density, along with a high compressive strength. All of the foams formulated with HFO-1336mzz (E) or (Z)–hexane and the HFO–hexane mixture show a very high compressive strength compared with the foam formulated only with hexane. This increase in compressive strength results not only from the high density of the foam but also from the uniform and small cells in the foam structure.

Due to their higher densities, the thermal conductivities of each of the PF OP foams are higher than those of the PFs. Though the thermal conductivities of the HFO blowing agents are lower than that of hexane, the foams formulated with them alone, or their mixtures with hexane, show high thermal conductivity. This may be attributed to evaporative loss of the low-boiling HFO-1336mzz during mixing in the foaming process. As a result, the quantity of blowing agents trapped in the resin matrix may be lower compared to the foams with hexane alone, resulting in an increase in the thermal conductivity of the foam.

The foam PF OP3, formulated with HFO-1336mzz (Z) and hexane, shows a relatively low density and thermal conductivity, along with a relatively high compressive strength, compared with the PF OP5 foam, which is formulated with a mixture of both HFOs and hexane. Though the compressive strength of PF OP5 is promising, its thermal conductivity is relatively high compared to that of PF OP3. Even though PF OP5 contains a combination of both HFO blowing agents, its conductivity is higher than that of the foam formulated with HFO-1336mzz (Z) and hexane. This difference in thermal conductivity may be due to the uneven distribution of the blowing agents in the resin matrix of PF OP5. The advantages of using the low-thermal-conductivity blowing agent are lost if the blowing agent is not adequately distributed and trapped within the resin matrix. Hence, the formulation was further modified by changing the surfactant concentration with the aim of achieving a better distribution of the reagents. The presence of the surfactant in the resin foaming process causes emulsification by reducing the surface tension between the resin–blowing agent interface [47]. This results in improved dispersion and stabilization of the blowing agent as small droplets throughout the resin mixture. Ethoxylated castor oil (Agnique CSO 30) surfactant was used in combination with polyethylene glycol 600 in all of the formulations. PEG has been used as both a surfactant [54] and a toughening agent [33,34] in many foaming reactions. Table 4 shows the modifications based on the PF OP5 formulation with variations in the concentrations of Agnique CSO-30 and PEG. In the first few samples, the concentrations of both Agnique CSO 30 and PEG were varied to check their effects on thermal conductivity and other properties. Later, the concentration of Agnique CSO 30 was kept constant, and the amount of PEG was varied. The blowing agent and catalyst quantities in each of these formulations were held constant. The properties of the resulting foams are listed in Table 5.

### 3.1. The Effect of PEG Concentration on the Density of the Foams

In the foam PF OP5, the total surfactant concentration (Agnique CSO 30 and PEG) is 8.8% with respect to the amount of resin, and the foam shows a density of 60 ± 3 kg/m^3^. In formulations PF OP5A and PF OP5B, the concentration of both surfactants is increased, i.e., the total surfactant concentration is increased to 12% and 14%, respectively. If the concentration of the surfactant is too high, the excess surfactant will be adsorbed onto the surface of the resin molecules, which will block the cross-linking reaction and also lower the volatilization of the blowing agents [47]. As a result, the density of the foam will increase. The densities of PF OP5A and PF OP5B are increased to 69 ± 3 kg/m^3^ and 77 ± 4 kg/m^3^, respectively, with an increase in the surfactant concentration. This increase in density may be due to the excess percentage of the surfactants and their impact on the cross-linking and evaporation of the blowing agents. In the formulations from PF OP5C through to PF OP5G, the concentration of Agnique CSO 30 was kept constant, and the amount of PEG was varied to study its ability to impart improvements in the mechanical strength of the foam. PEG was used as a toughening agent in the phenolic foams due to its ability to form bonds with the OH groups in the resin structure, thereby introducing long, flexible chains into the resin to impart toughness. Hu et al. [55] studied the effect of PEG on the mechanical properties, microstructure, thermal stability, and flame resistance of phenol– urea–formaldehyde foam. They found that the cell size, density, and compressive strength depend on the PEG concentration.

The foam PF OP5C was formulated with 6% (*w*/*w*) Agnique CSO 30 alone, which was the lowest surfactant concentration of all the samples. The density and thermal conductivity of PF OP5C are close to those of PF OP5, while its compressive strength is slightly higher, at 184 ± 20 kPa. This increase in compressive strength is due to the smaller cells in PF OP5C compared to those in PF OP5. In formulations PF OP5D to PF OP5G, the foam density decreased initially with the addition of PEG, reaching a minimum at 4% PEG, and started to increase with further additions of PEG. PF OP5C, formulated without PEG, showed a density of 61 ± 4 kg/m^3^, which decreased to 56 ± 4 and 49 ± 1 kg/m^3^ with 2% and 4% additions of PEG, respectively. With an increasing PEG concentration, the resin’s surface tension decreases, which facilitates emulsification, resulting in homogenous mixing of the blowing agents and facile expansion of the foaming mixture. The initial decrease in density is attributed to the surfactant behavior of the PEG toughening agent. PEG, along with Agnique CSO 30, helps to stabilize the HFO–hexane blowing agent bubbles and also decreases the resin’s surface tension, which facilitates efficient blending of the foamable mixture prior to expansion. Thus, the resulting foam exhibits a low density. The density starts to increase when the PEG concentration reaches 6% (*w*/*w*). The formulations PF OP5F and PF OP5G contain PEG concentrations of 12.4% and 12.8%, respectively, and exhibit densities of 58 ± 2 and 63 ± 1 kg/m^3^, respectively. At higher PEG concentrations, the hydroxyl groups in the PEG interact with the CH_2_OH groups present in the resin during the curing process at a high temperature to form an ether linkage. This limits the expansion rate of the foaming mixture, and gradually, the foam density increases as the concentration of PEG increases [34,55].

### 3.2. Effect of PEG on the Microstructure of the Foams

**Figure 6:**
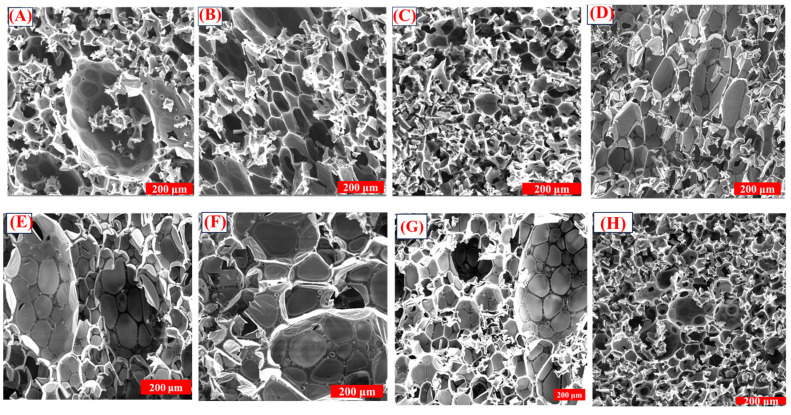
SEM images of the PFs prepared by changing the concentration of Agnique CSO 30 and PEG. (**A**) PF OP5, (**B**) PF OP5A, (**C**) PF OP5B, (**D**) PF OP5C, (**E**) PF OP5D, (**F**) PF OP5E, (**G**) PF OP5F, and (**H**) PF OP5G.

The changes observed in the thermal conductivity and compressive strength of the foams are better explained by microstructure analysis of the foams. Scanning electron microscopy was used to study the microstructure of the foams. The mean cell of each foam was calculated using ImageJ software by measuring the size of at least 100 cells, and the values are listed in Table 5. The SEM images of the foams formulated by varying the surfactant concentration are shown in Figure 6.

**Figure 7:**
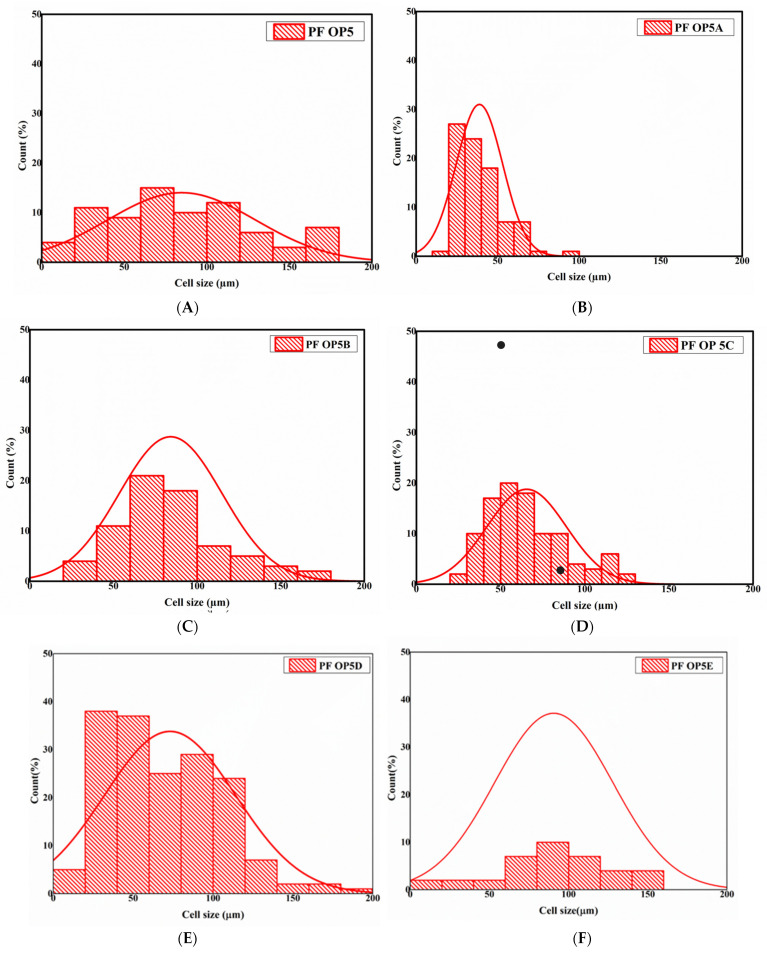

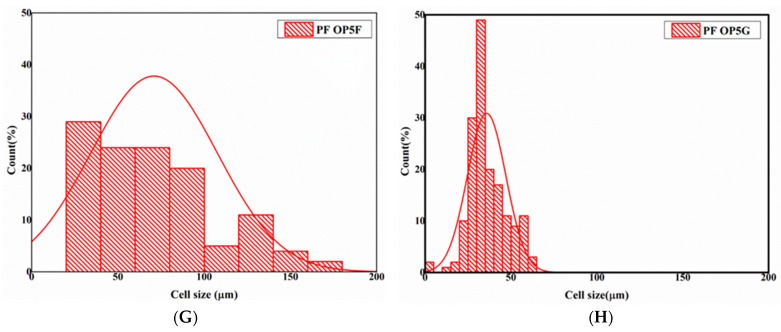
Cell size distribution of the foams formulated by changing the concentration of Agnique CSO 30 and PEG. (**A**) PF OP5, (**B**) PF OP5A, (**C**) PF OP5B, (**D**) PF OP5C, (**E**) PF OP5D, (**F**) PF OP5E, (**G**) PF OP5F, and (**H**) PF OP5G.

In formulations PF OP5, PF OP5A, and PF OP5B, the density increases with an increase in the surfactant concentration. Each of these foams shows a density-dependent cell size variation. The mean cell size of all three foams decreases as the density increases. PF OP5 had cell diameters ranging from 3 μm to 172 μm, with a mean cell size of 85 ± 14 μm. In PF OP5A, the cells became smaller, with a mean cell size of 66 ± 23 μm, whereas in PF OP5B, the cell size decreased further to 49 ± 14 μm. The cell size distribution of the foams is shown in Figure 7. The foam PF OP5C formulated without PEG contains elongated cells with a mean cell size of 65 ± 29 μm. The slight increase in compressive strength is attributed to the presence of small cells. With an increase in the surfactant concentration, the resin’s surface tension and viscosity will be reduced, resulting in efficient mixing of the reagents and better cross-linking within the resin structure. An appropriate surfactant concentration will offer much more stability to the blowing agents by ensuring a fine emulsion which aids their even distribution and expansion in a controlled manner during the foaming process. As a result, the cell structure becomes more uniform with smaller cells. In the formulations from PF OP5D to PF OP5E, the addition of PEG makes the foam expansion easier at lower concentrations, which leads to a decrease in density, facilitated by the expansion of the cells. With the addition of 2% PEG and 4% PEG, the mean cell size increases to 73 ± 40 μm and 93 ± 33 μm, respectively. After the optimum concentration, further addition of PEG leads to a more compact structure, resulting in an increase in density and a decrease in the mean cell size. The foams with 6.8% PEG show a mean cell size of 36 ± 10 μm, further confirming the toughening ability of PEG and the high compressive strength of the corresponding foams.

### 3.3. The Effect of PEG on the Compressive Strength of the Foams

The foam PF OP5 was formulated with 8.4% (*w*/*w*) surfactant and exhibited a density of 60 ± 3 kg/m^3^ and a compressive strength of 172 ± 6 kPa. The compressive strength of the foam is directly proportional to its density [56]. The increase in the compressive strength of PF OP5A and PF OP5B is due to the corresponding increase in their foam density. The small cells also contribute to the increase in the compressive strength of the foam. Like density, the compressive strength of the foams also decreases initially and then shows an increase with PEG concentration. Apart from their density, the addition of toughening agents to the foams can also influence their compressive strength. Toughening agents impart toughness by introducing flexibility into the resin structure. Adding PEG to the foam causes increased strength via the formation of ether linkages with the resin’s OH groups. Thus, the flexibility introduced by the long flexible resin–CH_2_–O–CH_2_–PEG chains into the resin’s structure provides a proper distribution of the blowing agents, surfactants, and catalysts in the resin matrix during foam formation, thereby toughening the phenolic foam and improving its mechanical strength.

**Figure 8:**
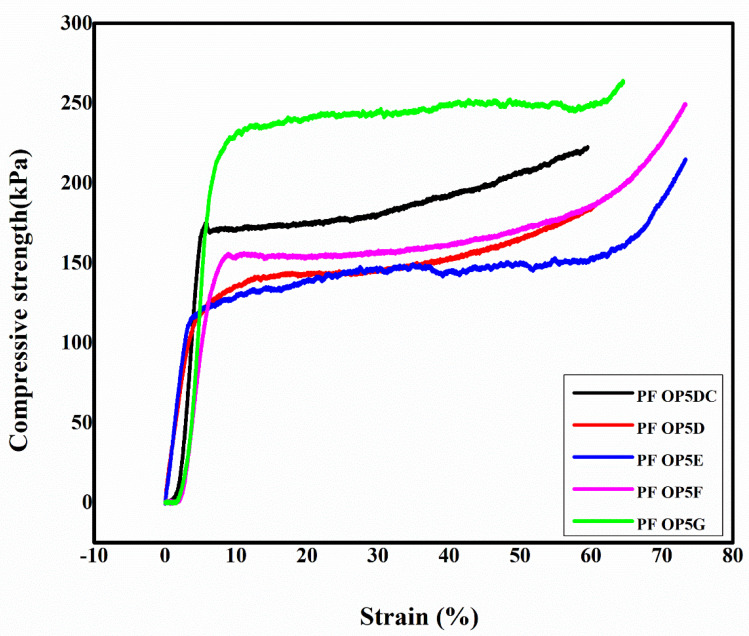
Stress–strain curves of PFs formulated by changing the concentration of PEG.

The compressive strength of PF OP5C formulated without PEG was 184 ± 20 kPa, which decreased to 127 ± 12 kPa and 129 ± 8 kPa in the foams formulated with 2% and 4% PEG. The decrease in compressive strength at a lower PEG concentration is due to the decrease in density as a result of the surfactant effect of PEG. The stress–strain curves of the PFs formulated by increasing the PEG concentration while maintaining the Agnique CSO 30 concentration as constant are shown in Figure 8.

**Figure 9:**
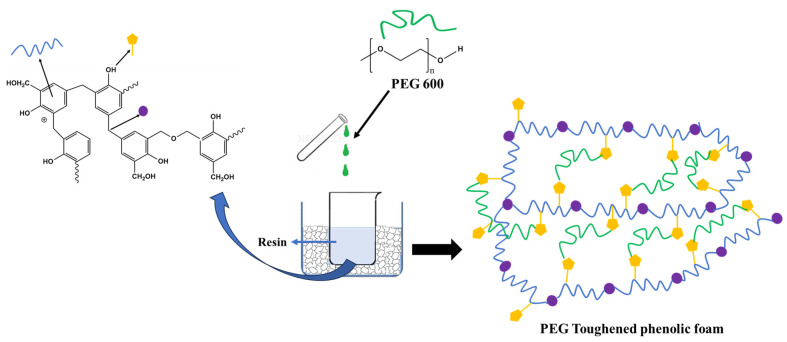
Toughening mechanism of PEG on phenolic foam.

In the formulations PF OP5F and PF OP5G, the PEG concentration was 6.4% and 6.8%, respectively. Density and compressive strength were observed to increase compared to the formulations with low PEG concentrations. The formulation PF OP5G, prepared with a total surfactant concentration of 12.8%, shows the lowest density, along with a high mechanical strength. This formulation provides good stability to the blowing agent emulsion in the resin and aids expansion in a controlled manner, resulting in a foam with a small and uniform cell structure. The cell size distribution becomes narrow with a mean cell size of 36 ± 10 μm. The uniform and small cells resulted in a very high compressive strength of 231 ± 7 kPa. The combined stabilization and toughening effect of PEG facilitates lowering the cell size and a uniform distribution of the blowing agents in PF OP5G. The toughening mechanism of PEG is shown in Figure 9. At a PEG concentration of 6.8%, the hydroxyl groups present in the PEG react with the –CH_2_OH functional groups in the resin, and long chains of PEG are introduced into the resin matrix, which gives higher flexibility under the applied stress. If the PEG concentration is increased further beyond 6.8% by weight, mixing becomes difficult due to agglomeration.

### 3.4. The Effect of PEG on the Thermal Conductivity of the Foams

The thermal conductivity of a foam depends on its density, the relative proportions of open and closed cells, cell size, and the distribution of the components within the foam. Foams with a low density and small, uniform, closed cells show a very low thermal conductivity, as they accommodate, trap, and distribute more blowing agent vapors throughout their structure. From Table 5, it is clear that the thermal conductivities of the PF OP5 foams mainly depend on the cell size and foam density. Foams with small cells and low density show lower thermal conductivities. PF OP5G and PF OP5D show the lowest thermal conductivities of all the foams. In PF OP5G, the small cell size, with diameters of 36 ± 10 um, contributes towards its low thermal conductivity, whereas in PF OP5D, its low density plays the major role.

Hence, the formulation PF OP5G is considered to be the best foam for thermal insulation applications, as it shows both a low thermal conductivity and a high compressive strength at a lower density.

### 3.5. Thermal Stability of the Foams Formulated by Changing the Concentration of PEG

**Figure 10:**
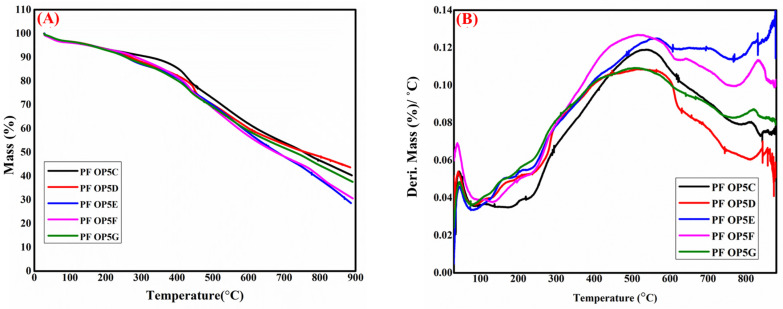
TGA (**A**) and DTG (**B**) graphs of phenolic foams formulated by changing the concentration of PEG.

The thermal stability of each of the PEG-toughened PFs was analyzed by thermogravimetric analysis (TGA). Figure 10 shows TGA and derivative thermograms of the foams toughened with various percentages of PEG, and their decomposition data are listed in Table 6. From the DTG curve (Figure 10B), it is clear that the phenolic foam with PEG shows an very similar degradation pattern with slight changes in degradation temperature and residual weight percentage. Both pure and PEG toughened foams exhibit three degradation steps.

In PF OP5DC, the first step occurs below 150 °C, followed by the second step between 150 and 250 °C, and the final step happens from 250 °C to 800 °C. The first step occurs mainly due to the evaporation of water, either present in the sample or formed by the condensation of phenol and formaldehyde, in addition to the unreacted formaldehyde and phenol within the foam. The second stage, between 150 and 250 °C, is also due to the degradation of the phenolic resin chains [57]. The maximum degradation occurs in the third step, which is caused by the breakage of the methylene linkages and ether bonds in the phenolic foam [34]. In PEG-toughened foams, the first degradation stage occurs below 100 °C, similar to pure PF, and the second degradation region is between 100 and 300 °C. The ether linkages (CH_2_–O–CH_2_) present in the PEG-toughened foams start to break near 200 °C, resulting in weight loss in this region. The initial 5% weight loss occurs in this second stage, and the T_5%_ values are listed in Table 6 The foam, PF OP5DC, formulated without PEG exhibited 5% weight loss at 157 °C. At very low concentrations, PEG stabilizes the foam, which is evident from the high thermal stability of PF OP5D during the first decomposition stage (between 100 and 200 °C). At higher temperatures, the PEG concentration significantly affects its thermal stability, as the T_30%_ (the temperature at which 30% weight loss occurs) values of all of the toughened foams are lower than that of the pure phenolic foam (PF OP5DC). These results indicate that the thermal stability of the PEG-toughened phenolic foams decreases with an increase in the PEG concentration. The PEG chains start to decompose around 200 °C, and the rate of decomposition increases with temperature, with the largest weight loss occurring in the range of 400–600 °C. Hu et al. [55] also reported a similar trend in the PEG-toughened phenol–urea–formaldehyde foams. The decomposition of the phenolic resin at a high temperature also facilitates the fastest weight loss in the third degradation stage. The temperature at which the maximum degradation occurred is shown in Table 6 The foams with the highest PEG concentrations (PF OP5F and PF OP5G) degraded faster than other toughened foams and the pure PF (PF OP5DC). The maximum degradation temperatures of PF OP5D and PF OP5DE are 549 °C and 562 °C, respectively, which are higher than those of all of the other foams. This contradictory trend may be due to the toughening effect of PEG at low concentrations. The residual weight percentage of all of the PEG-toughened foams except PF OP5D is lower than that of pure PF. The slightly higher residual weight percentage may be due to the better thermal stability offered by PEG at very low concentrations.

## 4. Conclusions

The effects of incorporating HFO blowing agents into phenolic foams have been investigated. Traditional blowing agents have a high ozone depletion potential (ODP), or a high global warming potential (GWP), resulting in the need for the development of alternative formulations. This study describes the preparation of phenolic foam using hydrofluoroolefin blowing agents such as HFO-1336mzz (Z) and HFO-1336mzz (E), which have zero ODP and a low GWP, and the toughening agent polyethylene glycol (PEG). Due to the low boiling points of the blowing agents, the formulations with HFO-1336mzz (Z) and HFO-1336mzz (E) alone resulted in foams with a high density due to the early evaporation of the blowing agents and the fast curing of the resin matrix. In order to maximize the availability and retention of HFO-1336mzz, the possibility of using a mixture of HFO-1336mzz blowing agents in combination with high-boiling hydrocarbon blowing agents was investigated using COSMO-RS modeling studies. Based on the promising COSMO-RS results, a series of PFs were formulated by combining HFO-1336mzz (E) and (Z) with higher-boiling hexane. PF OP3 (density: 63 ± 3 kg/m^3^) and PF OP5 (density: 60 ± 3 kg/m^3^), formulated with HFO-1336mzz (Z)–hexane and combined HFO–hexane mixtures, showed compressive strengths of 196 ± 6 kPa and 172 ± 6 kPa, respectively. It was found that a relatively small amount of HFO-1336mzz is sufficient to produce a strong phenolic foam. This study also analyzed the impact of toughening agents on the mechanical properties of the foams. The foams formulated with PEG toughening agent showed a concentration-dependent enhancement in their compressive strength. At a lower PEG concentration, the surfactant properties became prominent, and the resulting foams had a low density, whereas at higher concentrations, the toughening effect dominated, and the foam showed a high compressive strength, even at lower densities. With further optimization of the formulation, it has been demonstrated that the incorporation of HFO–hexane blowing agents, in combination with PEG toughening agents, can produce phenolic foams with improved thermal and mechanical properties. It is recommended that future studies focus on enhancing the percentage of the HFO blowing agents in the foam formulation after initial optimization by modeling studies using other possible organic blowing agents. The influence of other toughening agents, such as nanoparticles, fibers, and carbon materials, on foams formulated with HFO blowing agents also needs to be investigated to further optimize their thermal and mechanical performance.

## Figures and Tables

**Table 1:** Properties of blowing agents used in this study [50,51,52].

	HFO Blowing Agent		Hexane
IUPAC Name	Cis-1,1,1,4,4,4-hexafluoro-2-butene	Trans-1,1,1,4,4,4-hexafluoro-2-butene	N-hexane
Short Name	HFO-1336mzz (Z)	HFO-1336mzz (E)	Hexane
Flammability	Non-flammable	Non-flammable	Flammable
Boiling Point (°C)	33	7.5	69
Molecular Weight (g/mol)	164.05	164.05	86.18
Global Warming Potential	Low	Low	3
Ozone Depletion Potential	0	0	0
Thermal Conductivity—Liquid (W/m∙K)	0.0908 (at 20 °C)	0.0804 (at 25 °C)	0.124 (at 26.8 °C)
Thermal Conductivity—Vapor (W/m∙K)	0.0104 (at 20 °C)	0.0115 (at 25 °C)	0.165 (at 69 °C) [53]

**Table 2:** PFs formulated with HFO blowing agents.

	Resin Mass (g)	Agnique CSO 30 (g)	PEG (g)	Hexane (mL)	HFO-1336mzz (Z) (mL)	HFO-1336mzz (E) (mL)	MSA 70% (mL)
PF	50	2	2.4	7	0	0	4
PF OP1	50	2	2.4	0	0	7	4
PF OP2	50	2	2.4	0	7	0	4
PF OP3	50	2	2.4	6	1	0	4
PF OP4	50	2	2.4	6	0	1	4
PF OP5	50	2	2.4	5	1	1	4
PF OP6	50	2	2.4	4	1.5	1.5	4
PF OP7	50	2	2.4	4	1	2	4
PF OP8	50	2	2.4	2	4	1	4

**Table 3:** Properties of phenolic foams formulated using HFO and HFO–hexane mixture blowing agents.

Foam	Density (kg/m^3^)	Thermal Conductivity (W/m·K)	Compressive Strength (kPa)
PF	55 ± 2	0.036 ± 0.001	122 ± 8
PF OP1	Properties not measured due to lack of foam expansion		
PF OP2	99± 5	0.040 ± 0.001	306 ± 7
PF OP3	63 ± 3	0.036 ± 0.001	196 ± 6
PF OP4	70 ± 2	0.042 ± 0.001	216 ± 8
PF OP5	60 ± 3	0.037 ± 0.001	172 ± 6
PF OP6	82 ± 2	0.038 ± 0.001	289 ± 6
PF OP7	80 ± 6	0.039 ± 0.001	298 ± 7
PF OP8	94 ± 4	0.041 ± 0.001	330 ± 11

**Table 4:** PFs formulated with HFO–hexane mixtures, varying the surfactant concentration.

	Resin Mass (g)	Agnique CSO 30 (g)	PEG (g)	Hexane (mL)	HFO-1336mzz (Z) (mL)	HFO-1336mzz (E) (mL)	MSA 70% (mL)
PF OP5	50	2	2.4	5	1	1	4
PF OP5A	50	3	3	5	1	1	4
PF OP5B	50	4	3	5	1	1	4
PF OP5C	50	3	0	5	1	1	4
PF OP5D	50	3	1	5	1	1	4
PF OP5E	50	3	2	5	1	1	4
PF OP5F	50	3	3.2	5	1	1	4
PF OP5G	50	3	3.4	5	1	1	4

**Table 5:** Properties of PFs prepared by changing the concentration of Agnique CSO 30 and PEG.

	Density (kg/m^3^)	Thermal Conductivity (W/m·K)	Compressive Strength (kPa)	Mean Cell Size (μm)
PF OP5	60 ± 3	0.037 ± 0.001	172 ± 6	85 ± 14
PF OP5A	69 ± 3	0.037 ± 0.001	206 ± 5	66 ± 23
PF OP5B	77 ± 4	0.037 ± 0.001	225 ± 5	49 ± 14
PF OP5C	61 ± 4	0.037 ± 0.001	184 ± 20	65 ± 29
PF OP5D	56 ± 4	0.035 ± 0.001	127 ± 12	73 ± 40
PF OP5E	49 ± 1	0.036 ± 0.001	129 ± 8	93 ± 33
PF OP5F	58 ± 2	0.036 ± 0.001	154 ± 5	75 ± 41
PF OP5G	63 ± 1	0.035 ± 0.001	231 ± 7	36 ± 10

**Table 6:** TGA data for pure and PEG-toughened phenolic foams.

Samples	T_5%_ (°C)	T_30%_ (°C)	T_max_ (°C)	Residual Weight (%) at 885 °C
PF OP5C	156.9	524.8	537.4	40.6
PF OP5D	159.4	501.2	549.3	43.6
PF OP5E	156.9	499.2	562.3	28.8
PF OP5F	151.0	489	516	31.2
PF OP5G	155.7	490.5	498.1	38.0

## Data Availability

Data are contained within the article.
